# Spatiotemporal association of rapid urbanization and water-body distribution on hemorrhagic fever with renal syndrome: A case study in the city of Xi’an, China

**DOI:** 10.1371/journal.pntd.0010094

**Published:** 2022-01-10

**Authors:** Li Shen, Minghao Sun, Xiao Wei, Yao Bai, Qingwu Hu, Shuxuan Song, Boxuan Gao, Weilu Zhang, Jifeng Liu, Zhongjun Shao, Kun Liu

**Affiliations:** 1 School of Remote Sensing and Information Engineering, Wuhan University, Wuhan, People’s Republic of China; 2 Department of Epidemiology, Ministry of Education Key Lab of Hazard Assessment and Control in Special Operational Environment, School of Public Health, Air Force Medical University, Xi’an, People’s Republic of China; 3 Department of Infectious Disease Control and Prevention, Xi’an Center for Disease Prevention and Control, Xi’an, People’s Republic of China; Saudi Ministry of Health, SAUDI ARABIA

## Abstract

Hemorrhagic fever with renal syndrome (HFRS) is a zoonosis characterized by clinical features of high fever, hemorrhage, and renal damage. China has the largest number of HFRS cases worldwide, accounting for over 90% of the total reported cases. In this paper, we used surveyed HFRS data and satellite imagery to conduct geostatistical analysis for investigating the associations of rapid urbanization, water bodies, and other factors on the spatiotemporal dynamics of HFRS from year 2005 to 2018 in Xi’an City, Northwest China. The results revealed an evident epidemic aggregation in the incidence of HFRS within Xi’an City with a phenomenal fluctuation in periodic time series. Rapid urbanization was found to greatly affect the HFRS incidence in two different time phases. HFRS caused by urbanization influences farmers to a lesser extent than it does to non-farmers. The association of water bodies with the HFRS incidence rate was found to be higher within the radii of 696.15 m and 1575.39 m, which represented significant thresholds. The results also showed that geomatics approaches can be used for spatiotemporally investigating the HFRS dynamic characteristics and supporting effective allocations of resources to formulate strategies for preventing epidemics.

## Author summary

Hemorrhagic fever with renal syndrome (HFRS) is an infectious disease transmitted by the infected reservoir hosts, which chiefly include rodents, shrews, and moles. The mechanism of HFRS infection is determined by a complex interplay of various natural and socioeconomic factors. Previous studies have identified a close relationship between urbanization processes and HFRS transmission dynamics. However, the relationship involves a dual response and has not yet been quantitatively identified. This research aimed to spatiotemporally characterize how rapid urbanization affects the dynamics of HFRS. In addition, the role of water bodies, vegetation, rainfall, and occupation types in epidemic transmissions has also not been adequately investigated at the interurban scale over a long time period. Therefore, we analyzed the spatiotemporal association between the distributions of water bodies and HFRS infections and also investigated the interactions between urbanization, water body, vegetation, rainfall, and different occupation types by using multi-source data through both traditional statistical and geospatial techniques. Our work revealed biphasic associations among HFRS infection with both rapid urbanization and distribution of water bodies. Hence, it can provide useful information to formulate effective strategies for the prevention and control of HFRS.

## Introduction

Hemorrhagic fever with renal syndrome (HFRS) is a zoonosis caused by Hantaviruses (family Bunyaviridae) [1–3]. This disease is clinically characterized by fever, hemorrhage, and renal dysfunction, with a fatality rate of 0.5%–40% depending on the specific viral strain involved [4]. Hantaviruses could be transmitted from the infected reservoir hosts to humans when the latter comes in contact with the contaminated saliva, urine, or excreta or by inhaling virus-contaminated aerosols [5]. People living in poor conditions and working in crowded places are more vulnerable to hantaviruses because they are frequently exposed to the body fluids and excreta of infected rodents [5,6]. HFRS has been recognized as a severe global public health problem as approximately 60,000–100,000 HFRS cases are reported annually by more than 70 underdeveloped countries in Asia and Europe [7,8]. Since the 1950s, China has reported over 1.56 million HFRS cases with a fatality rate of nearly 3.0%, accounting for 90% of the total reported cases worldwide [9]. Accordingly, Chinese government has carried out some effective initiatives for the prevention and control of HFRS, such as tracking changes in the habitats of rodents, sharing epidemic information with the general public, improving living and working conditions of its people, and implementing free vaccination drives. However, HFRS still remains one of the top nine communicable diseases in mainland China, and it continues to reemerge in certain epidemic-prone areas, especially in the northeastern and northwestern provinces, posing a serious long-term danger to public health and safety.

HFRS cases in Shaanxi Province mainly involve two serotypes of Hantaviruses, HTNV and SEOV, the reservoir hosts of which are *Apodemus agrarius* and *Rattus norvegicus*, respectively [10]. The former is the dominant species in Shaanxi Province and are typically found in rural fields. The latter normally resides in residential areas [11]. Scientists have made significant efforts to understand how environmental conditions (e.g., precipitation, temperature, and landscape) and anthropological factors (e.g., urbanization, agricultural activity, and vaccination) alter rodent reservoir dynamics and then trigger HFRS outbreaks [12]. Research has not only emphasized the association between HFRS incidence and geophysical conditions [13,14], but also noted multiple processes linking anthropogenically driven environmental changes (e.g., land-use changes) and HFRS dynamics in recent years [15,16]. Previous research has shown that the geographic distribution of HFRS is significantly associated with the artificial area, cropland, and precipitation and indicated that the incidence of HFRS first increases to a peak value and then gradually decreases or plateaus [11]. In the city of Xi’an, the rodent density has been recognized as a major factor that influences HFRS outbreaks, while rainfall and temperature have been identified as indirect factors [17]. However, recent research has shown that the epidemic areas of HFRS have expanded to urban–rural fringe regions in some cities, including Xi’an. However, the precise reasons for this phenomenon have not been identified yet because of the complex and multifactorial rodent reservoir dynamics [15]. A possible explanation is that urbanization in Xi’an can impact the population dynamics of the rodent reservoir; however, no consensus has yet been reached on the exact relationships between urbanization and infectious diseases because of the contrasting effects [18]. Urbanization may increase the risk of HFRS emergence and transmission before peak is reached after which the HFRS incidence reduces owing to improved environmental conditions and better fundamental infrastructure [5]. In addition, existing literature suggests that rodent species in built-up area and water bodies can affect the risk of HFRS occurrence [19]. Therefore, it is necessary to improve our understanding of how anthropogenic activities, natural environment, and HFRS are interrelated. To understand this interrelationship, we have conducted our study in the city of Xi’an. Very few studies have focused on quantitatively detecting the complex relationship between urbanization and HFRS transmission from a spatiotemporal perspective. Moreover, the roles of water bodies, vegetation, rainfall, and occupation type in epidemic transmissions have also not been adequately investigated at an interurban scale over a long time period.

The present work aims to spatiotemporally characterize how rapid urbanization and distribution of water bodies, vegetation, rainfall, and occupation type relate to HFRS dynamics in Xi’an City. A retrospective epidemiological study was conducted based on geostatistical analyses using an integrated geodatabase (surveillance of HFRS infections, satellite data, and vector data) from 2005 to 2018 as the input information. From a spatial perspective, we established a hierarchical index system to quantify the urbanization level in Xi’an City for a comprehensive evaluation of its association with the incidence of HFRS. From a temporal perspective, the variations in the urbanized areas extracted from satellite imagery were compared with the historically surveyed HFRS cases for interpreting 14-year continual trends on HFRS dynamics. Furthermore, we performed circular buffer analyses to understand the spatiotemporal association between the distribution of water bodies and HFRS infections. Finally, we detected the interactions between urbanization, water body, vegetation, rainfall, and occupation types to theoretically improve our understanding of how multiple factors co-influence the epidemic dynamics of HFRS from a spatiotemporal perspective.

## Materials and methods

### Study area

This study was carried out in Xi’an, the capital city of Shaanxi Province in northwestern China (107°24’–109°24’E, 33°25’–34°27’N) (S1 Fig). Xi’an consists of 11 administrative districts and two counties and has a total area of 10,752 km^2^. In 2019, its permanent population was 10.037 million. It has a semi-humid continental monsoon climate with a dense hydrographic network of 54 rivers comprising 98.46% of the Yellow River Basin. Since China reported its first HFRS case in Huxian County in 1956, Shaanxi Province has become one of the major centers of HFRS endemic outbreak in China. According to the Chinese Center for Disease Control and Prevention (CCDC), Shaanxi Province has been reporting 1,000 HFRS human cases every year since the past decade, with a peak incidence of over 3,500 cases in 2012 [8]. Within Shaanxi Province, Xi’an is the most vulnerable city to HFRS outbreaks [20–22]. Although the high-risk population in the entire region has been vaccinated more than ten years ago, Xi’an still greatly suffers from HFRS endemic outbreaks, with a noticeably higher incidence rate than that of other regions in China. In addition, the spatiotemporal characteristics of HFRS incidence in Xi’an exhibit clear clustered patterns moving from the rural areas to the suburban areas. These results shows that it is imperative to investigate how the risk of HFRS is spatiotemporally related with different natural and socioeconomic factors in Xi’an and why it is considered a typical high-risk area for HFRS epidemic in China.

### Data collection and management

Our research data include the surveyed HFRS cases, satellite imagery, vector boundaries, and associated socioeconomic census data. The surveyed HFRS cases were provided by Center for Disease Control and Prevention of Xi’an and included 12,945 cases covering a span of 14 years from 2005 to 2018. The data contain abundant information about the sex, age, occupation, address, and other relevant details of the patients. We converted each text residential address into a spatial coordinate point to be geographically overlaid with the corresponding vector map of the study area and then assigned the matching attributes to those converted geographical points to construct a geodatabase.

In addition, we utilized the Landsat satellite imagery to extract information about the urbanized areas and water bodies (rivers, lakes, and artificial waters) in Xi’an. After previewing all the available imagery from the United State Geological Survey (USGS; https://earthexplorer.usgs.gov/) online archive, we acquired 45 Landsat-7 and Landsat-8 multiband images with cloud cover of less than 10% for the period of 2005 to 2018. Next, we obtained information on the urban built-up areas and distribution of water bodies by implementing the supervised classification approach based on ENVI 5.3 software with an accuracy of over 90%, which can well satisfy the requirements for the subsequent analysis. Normalized differential vegetation index (NDVI), an indicator of vegetation growth and spatial difference, was also calculated from the Landsat imagery using ENVI 5.3 software. The rainfall data with a spatial resolution of 1 km and a temporal resolution of 1 month were acquired from the National Earth System Science Data Center, National Science & Technology Infrastructure of China (http://www.geodata.cn), in the form of a net.CDF file. Base map and vector boundaries of Xi’an City were obtained from the basic geographic database in National Catalogue Service For Geographic Information of China (https://www.webmap.cn) with the licensing agreement (https://www.webmap.cn/main.do?method=otherService&clickFlag=service).

**Fig 1:**
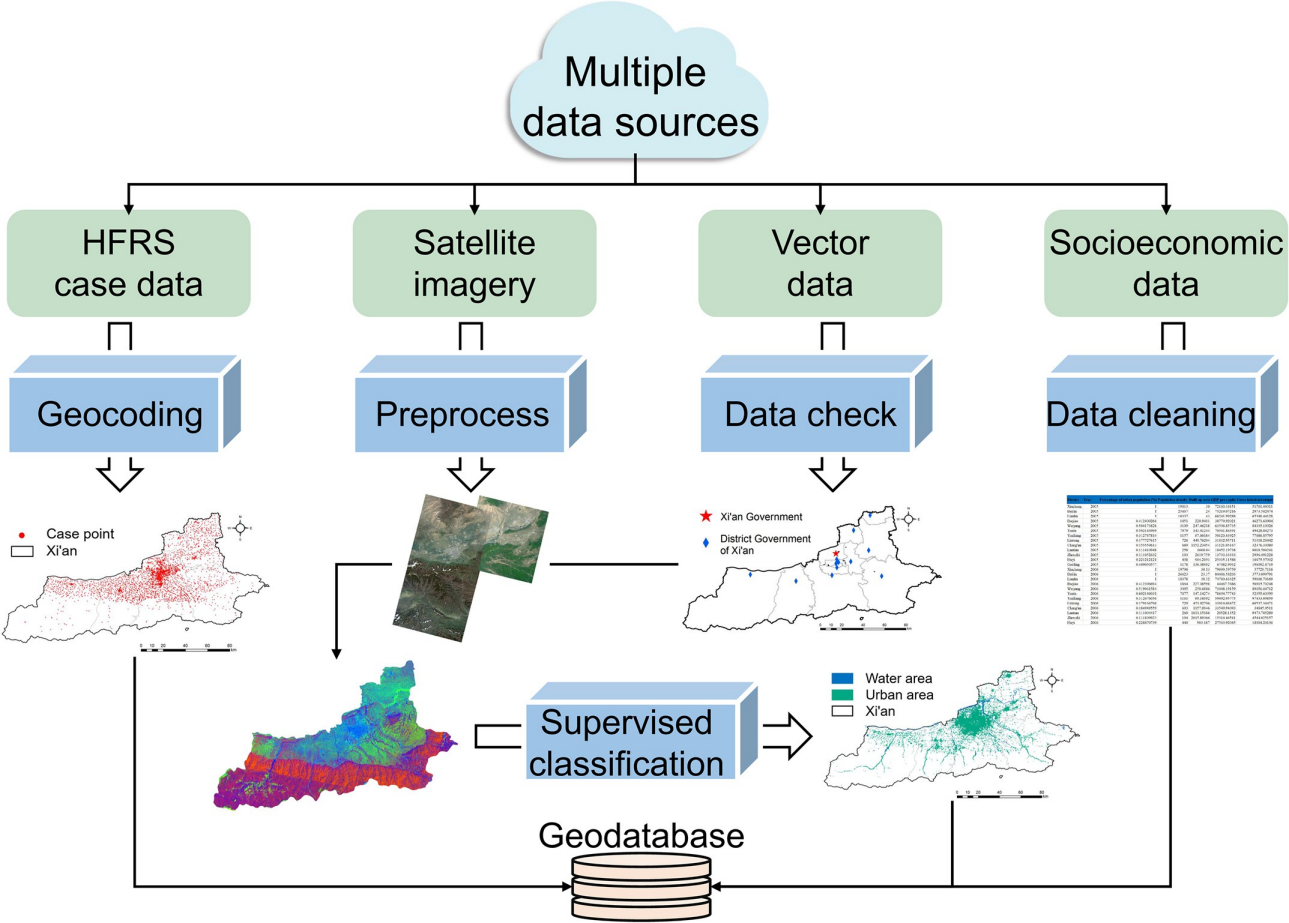
Multiple data sources and establishment of a geodatabase. The maps in the figure were produced in ArcGIS 10.8 (ESRI, Redlands, CA, USA) using shape files representing Xi’an City which were obtained from the basic geographic database in National Catalogue Service For Geographic Information of China (https://www.webmap.cn/mapDataAction.do?method=forw&resType=5&storeId=2).

The socioeconomic census data including two grades and nine categories of indicators were obtained from the Statistical Yearbook of Xi’an Municipal Bureau of Statistics. These statistical data were carefully examined and cleaned to make sure the format is suitable for the subsequent analysis. Finally, after integrating the HFRS spatial data, vector boundaries, image-derived urbanized areas, and water bodies, we used ArcGIS 10.8 software to establish a geospatial database for further analysis (Fig 1).

### Geospatial statistical analysis

#### Spatiotemporal pattern analysis

**Fig 2:**
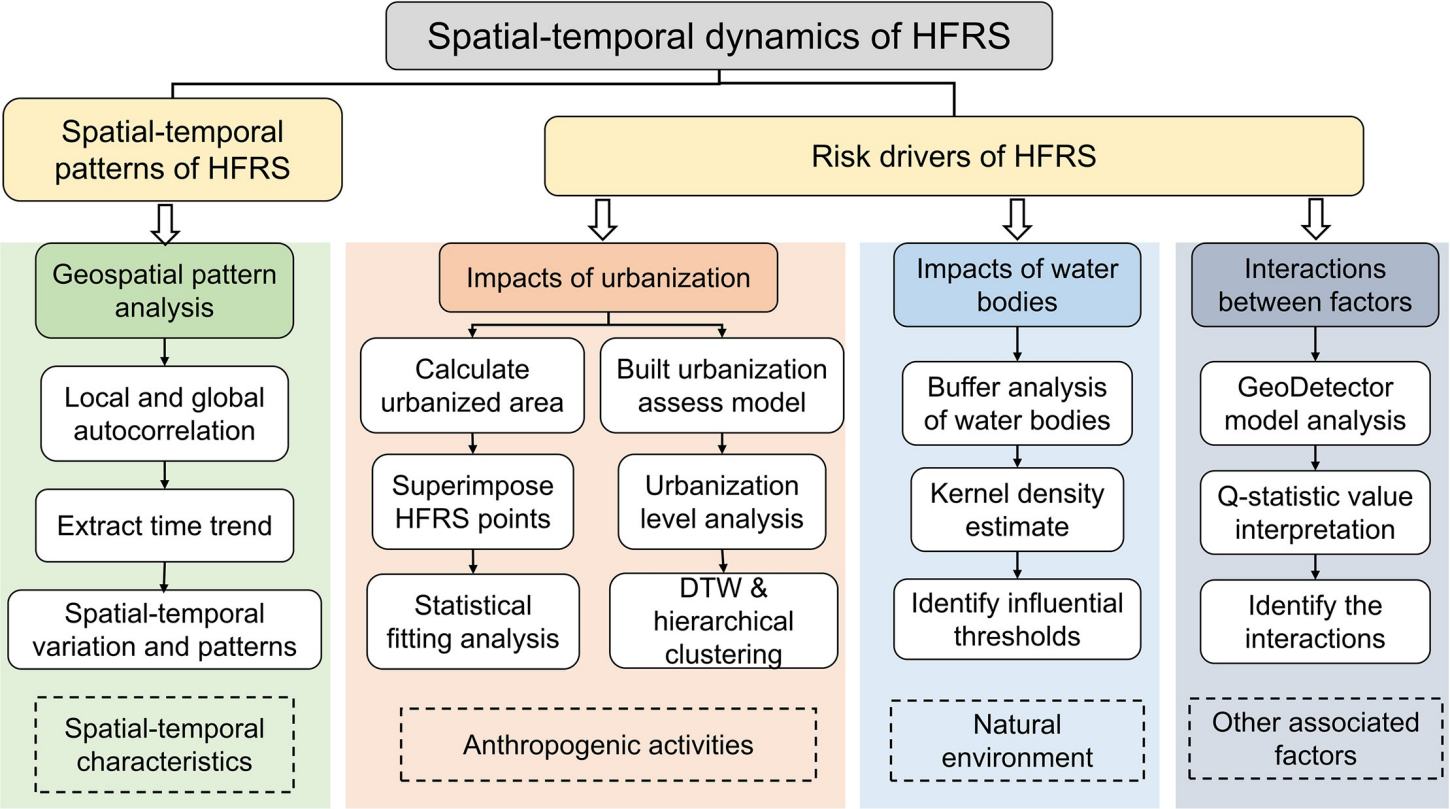
Overall methodology flowchart.

Our research has three main objectives: (i) to extract the spatiotemporal patterns of HFRS incidence for the period of 2005–2018, (ii) to characterize the association of rapid urbanization and distribution of water bodies with the HFRS incidence, and (iii) to investigate interactions between water bodies, NDVI, rainfall, occupation types, and urbanization on the HFRS incidence. The methodology flowchart is shown in Fig 2.

Firstly, we applied geospatial statistics, including both global autocorrelation (Moran’s I) and local autocorrelation (LISA) analyses, to detect the spatial dynamic patterns (random, dispersed, or clustered) of the HFRS incidence across Xi’an City for each year from 2005 to 2018. Moran’s I is a global pattern analysis used for the overall evaluation of the autocorrelation status, while LISA is used to identify the clusters and outliers from a local perspective [23–25]. In addition, fitting analysis was conducted to capture the temporal variations in the HFRS incidence between 2005 and 2018 based on a monthly trend line.

#### Spatiotemporal analysis of the association of urbanization with HFRS

**Fig 3:**
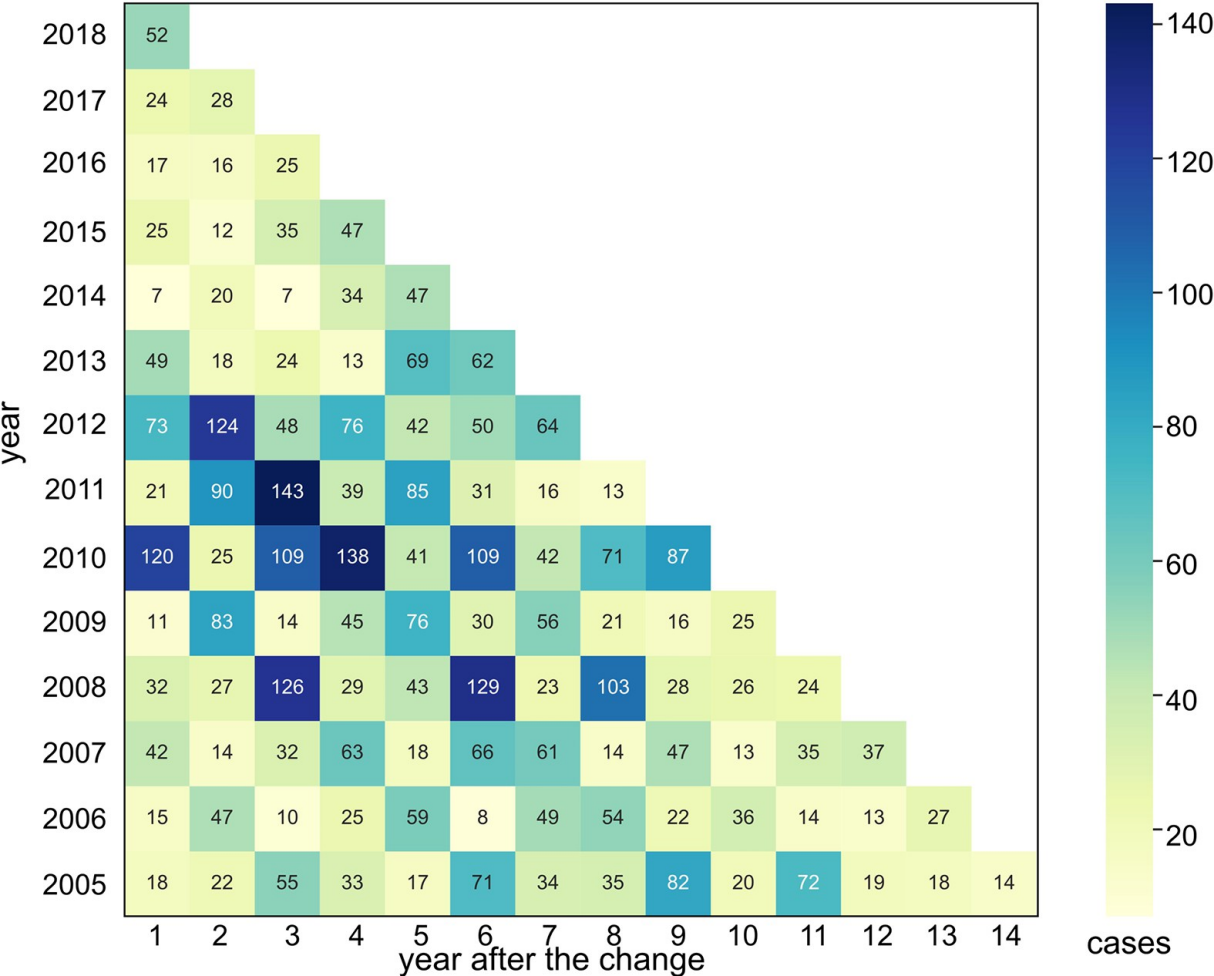
Statistical calculation of HFRS cases within the changed urbanization area from 2005 to 2018 for Xi’an.

An urbanized area is generally defined as an impervious surface that can be extracted from satellite imagery by applying remote sensing methodologies [13]. We compared the urbanized area for each year with that of the previous year to calculate the variation in urbanization. Then, we categorized these changes into three major types: (i) change from a nonurban area to an urban area, (ii) change from an urban area to a nonurban area, and (iii) an unchanged area. Variation in urbanization for these three types were saved as vector data and imported into the geodatabase. We focused on analyzing the first category, i.e., change from a nonurban area to an urban area, which represents the most prominent urbanization process. The HFRS case points were overlaid on the vector data layer of the nonurban to urban area using the GIS software to calculate how many HFRS cases located within the vector boundary compared to that in the following year (Fig 3) and obtained the expected variation, which can be effectively demonstrated in a continuous trend line using quadratic function fitting.

To better interpret the association of rapid urbanization with the HFRS incidence, we also quantitatively measured the urbanization level in Xi’an using a composite index developed by considering various socioeconomic factors such as urban population, financial condition, and social development. We selected nine specific indicators in two hierarchical grades to quantify the urbanization level by integrating various demographic, economic, and social aspects of urban development (Table 1). To eliminate the influence of magnitude and scale difference, the original socioeconomic census data was first standardized following Equation (1) and then used to calculate the index. Next, we applied the entropy weight method to determine the weight of each indicator by reserving important values and avoiding subjective deviation (Table 1) [26]. The details on the entropy weight calculation can be found in the Supporting Information.

**Table 1:** Comprehensive evaluation indicators of urbanization level.

First-grade indicator	Second-grade indicator	Weight
Urbanization of population	Percentage of urban population (%)	0.069
	Population density (persons/km^2^)	0.075
Urbanization of land use	Built-up area (km^2^)	0.132
Economic urbanization	GDP per capita (CNY)	0.119
	Gross industrial output per capita (CNY)	0.101
	Total retail sales of consumer goods (CNY)	0.129
	Proportion of the added value of the tertiary industry to GDP (%)	0.158
	Total investment in fixed assets (CNY)	0.065
Social urbanization	Number of doctors per 10,000 people (persons)	0.154

GDP: Gross domestic product; CNY: Chinese Yuan.

The weight matrix was multiplied by the interval data to obtain the final overall urbanization index set for the 13 counties and districts in Xi’an for each year from 2005 to 2018. Similarly, we obtained a fitting scatter plot based on the quadratic function to describe the relationship between the urbanization level and the HFRS incidence rate for each county and district. Because the HFRS incidence can be considered to change in the univariate time series, we implemented the dynamic time warping (DTW) method to retrieve the similarity of the urbanization associations with HFRS for each district and county in Xi’an and then classified the associations into four types of clusters [27]. The DTW distance was computed between every two curves in different districts and counties to build a DTW distance matrix. Next, the hierarchical clustering algorithm was used to divide the curves into different clusters based on the obtained matrix of similarity.

#### Spatiotemporal analysis of the association of water bodies with HFRS

**Fig 4:**
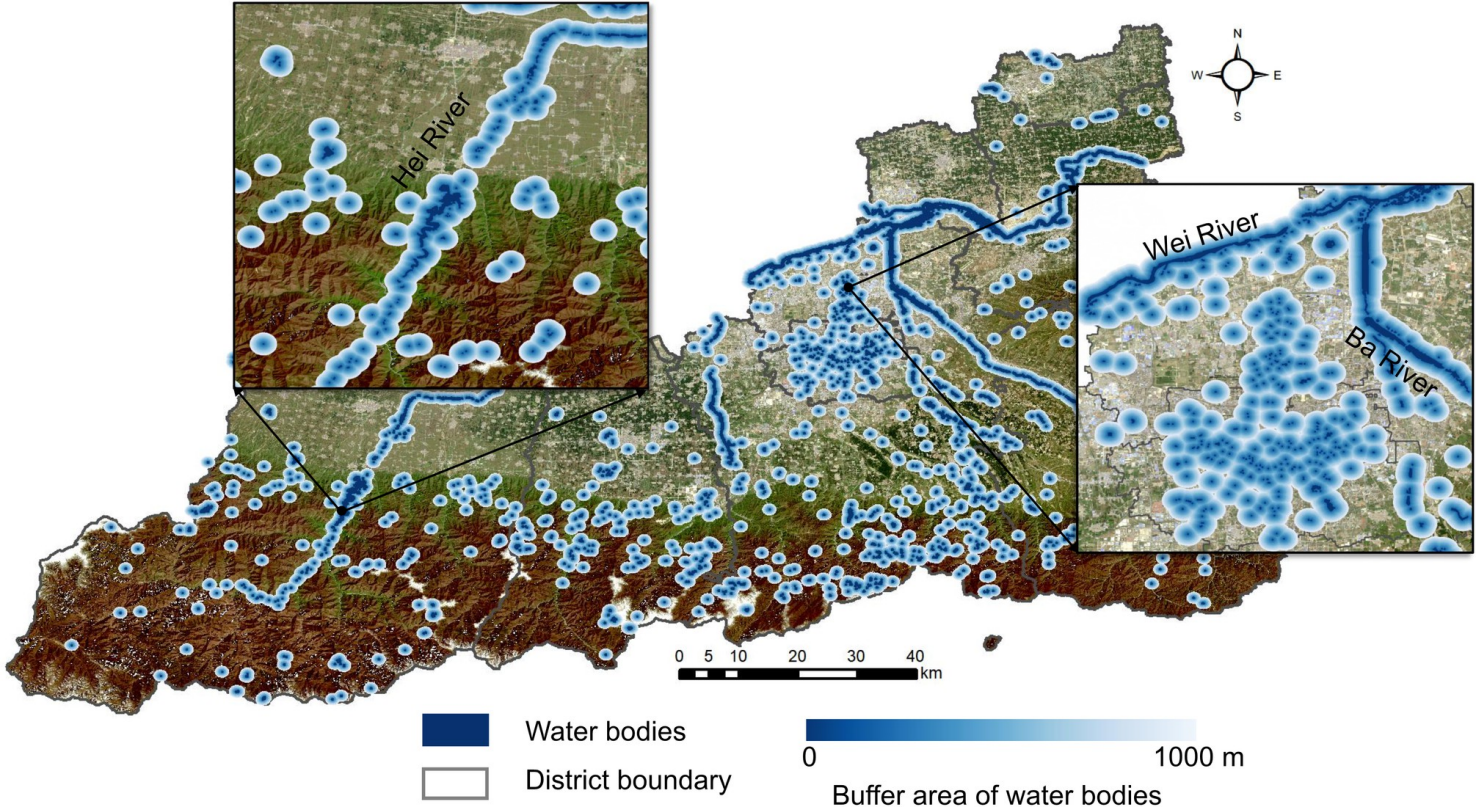
Buffer analysis for water bodies in Xi’an City. The figure was produced in ArcGIS 10.8 (ESRI, Redlands, CA, USA) using shape files representing Xi’an City which were obtained from the National Catalogue Service For Geographic Information of China (https://www.webmap.cn/mapDataAction.do?method=forw&resType=5&storeId=2).

To spatiotemporally investigate the associations of water bodies with the HFRS incidence, we conducted a buffer analysis for each year based on the ArcGIS 10.8 software (Fig 4). Firstly, the buffer zones were extracted at different distances from the vector features of water bodies across Xi’an. Then, the number of HFRS cases and density in each buffer zone were calculated. The maximum and minimum intervals of the buffer distance were set as 1,000 m and 50 m, respectively. To detect the characteristic distance threshold of water bodies affecting the HFRS incidence, we computed the ratio of HFRS density by taking the HFRS cases into account within and outside the buffer zones. The final comparative trends of the characteristic distance thresholds were fitted and plotted for each year.

In addition, to quantitatively explore the characteristic thresholds, we calculated the distances between each HFRS case point and the nearest water body for all the 14 years and then used the kernel density estimate (KDE) to determine the peak thresholds. As a nonparametric test method, the KDE has been widely used to examine the first-order properties of a spatial process and to produce a smooth continuous probability density curve in one or more dimensions [28]. In the present study, the spatial relation between the distribution of HFRS cases and water bodies was measured by computing the Euclidean distance from each case point to the vector edge of water bodies, which was arranged based on the onset time. Next, after smoothing the observations of distance with a Gaussian kernel, we extracted the peak points of the KDE curve. A bivariate distribution of HFRS cases was established by considering the age and sex of patients. Variations in the annual peak values were deleted to explore the possible cycles. All these operations were performed on the ArcGIS 10.8 platform and Jupyter Notebook along with the Python libraries including seaborn 0.11.1 and SciPy 1.6.0.

#### Analysis of the interactions between urbanization and other factors on HFRS

To investigate whether rapid urbanization and other factors including water bodies, climatic conditions, and vegetation impact the HFRS incidence, we applied the GeoDetector model—an advanced statistical method—to quantitatively capture such interactive associations. This model was proposed by Wang et al. in 2010 based on the theory of spatial stratified heterogeneity (SSH) [29]. The GeoDetector model enables researchers to quantify the interactions between two explanatory variables, *X*_*1*_ and *X*_*2*_, and a response variable, *Y*, which can be accomplished by the *q*-statistic. In our study, *Y* is the number of HFRS cases in a statistic unit, and urbanization, water bodies, rainfall, and NDVI are all recognized as explanatory variables. Statistic units are composed of 5-km grids covering the entire city of Xi’an. Because these explanatory variables are numerical, we used the Natural Breaks tool in the ArcGIS 10.8 software to transfer them to categorical variables. In addition, the relation between urbanization and different types of occupation on the HFRS incidence was explored by *q*-statistic analysis. The details about the *q*-statistic calculation can be found in the Supporting Information.

## Results

### Spatiotemporal patterns of HFRS

The global spatial autocorrelation statistically demonstrated an overall clustered pattern of the HFRS incidence across Xi’an City from 2005 to 2018 (S1 Table). The spatial dynamic of the HFRS incidence in Xi’an is presented in S2 Fig. It can be clearly seen that most districts with high incidence were distributed in the middle region of Xi’an. Similarly, the results of local autocorrelations also revealed that the most significant high-high clusters, which refer to aggregations of high incidence, were concentrated in the south-central region of Chang’an District (S2 Fig). The incidence of HFRS in Lintong, Gaoling, and Baqiao districts in the northwestern region of Xi’an was usually lower than that in the other districts during most part of the study duration. The disparity between the incidences within these two regions is possibly owing to their distinct urban development conditions and population densities. For the temporal distribution of HFRS incidence in Xi’an City, the overall variation of cyclical fluctuations was obtained from the fitted time trend line shown in S3 Fig. A high endemic outbreak of HFRS infection may occur each year at two specific time periods: June–July and November–January (next year). Furthermore, a continually increasing and then a decreasing trend in the number of HFRS cases can be observed for the periods of 2005–2010 and 2010–2018, respectively. The peak year for the incidence was 2010, mainly owing to the high incidence of HFRS infection in November.

### Spatiotemporal association of urbanization with HFRS

**Fig 5:**
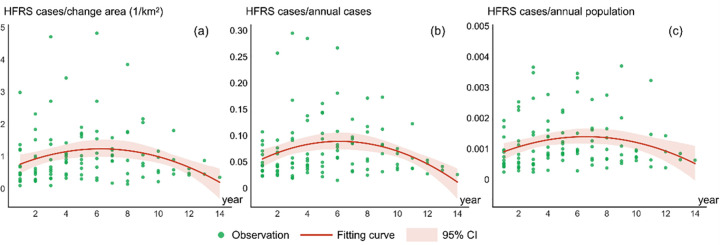
Relationships between urbanization and incidence rate of HFRS. Number of HFRS cases (a) divided by the transformed urbanized area in Xi’an, (b) divided by its total annual population within the changed area in Xi’an, and (c) divided by the total HFRS cases within the changed area in Xi’an.

The descriptive statistics for HFRS cases within the changed urbanization area from 2005 to 2018 for Xi’an is presented in Table 1. Since rapid urbanization can cause significant spatiotemporal associations with HFRS infections, the first row with numbers 1, 2, and 3 and so on in Table 1 represents the number of years a nonurban area takes to change into an urbanized area. Numbers the first column (2005, 2006, and 2007 and so on) signify the specific year. A comparative analysis was performed by considering the following three factors: (i) the ratio of HFRS cases to the newly transformed urbanized area; (ii) the ratio of HFRS cases within an urbanized area to the total number of annual HFRS cases; and (iii) the ratio of HFRS cases within an urbanized area to the total annual population within that urbanized area. The associations in Fig 5(A), 5(B) and 5(C) evidently show a continually increasing and then a decreasing tendency, as is evident from the plotted fitting trend lines. The association of urbanization processes with the HFRS incidence in Xi’an significantly peaked in the sixth year because of the highest ratio of HFRS cases to that in the transformed urbanized areas, the total number of annual HFRS cases, and the total annual population in that year. Hence, it is clear that the city of Xi’an shows two characteristic phases for the association of urbanization with HFRS infection during the entire urbanization period. In the first phase, urbanization progressively increases the rate of HFRS infection, while in the second observed after six years, the HFRS incidence gradually decreased to approach the lower level.

**Fig 6:**
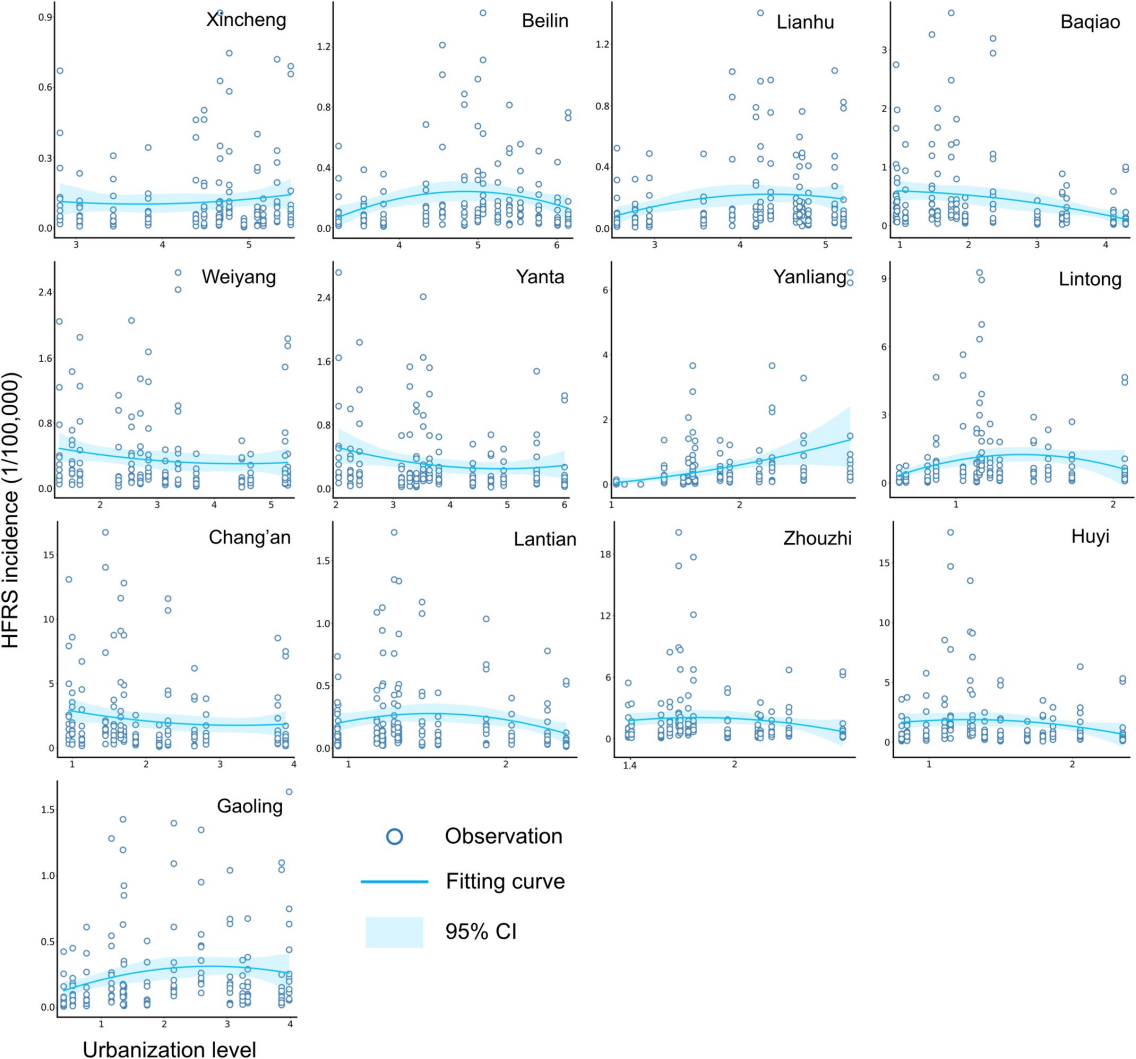
Scatter plot between incidence rate and urbanization level for 13 counties and districts of Xi’an City.

In addition, we examined the scatter plots between the developed urbanization index and the HFRS incidence rate for the 13 counties and districts of the city of Xi’an from 2005 to 2018 (Fig 6). While the urbanization level for each county and/or district of Xi’an increased during the observation period, the rate of HFRS incidence changed differently over time. For most regions such as Weiyang and Lantian districts, the fitting curve showed a peak value, which not only suggested an epidemic outbreak in that year, but also signified how urbanization level impacted the HFRS epidemic. Fig 6 also confirms the previously found two characteristic phases for revealing the association of rapid urbanization with the rate of HFRS incidence. However, some counties or districts such as Yanliang and Baqiao demonstrate merely one phase, either with an increasing or a decreasing trend, which can be determined based on its urbanization status.

**Fig 7:**
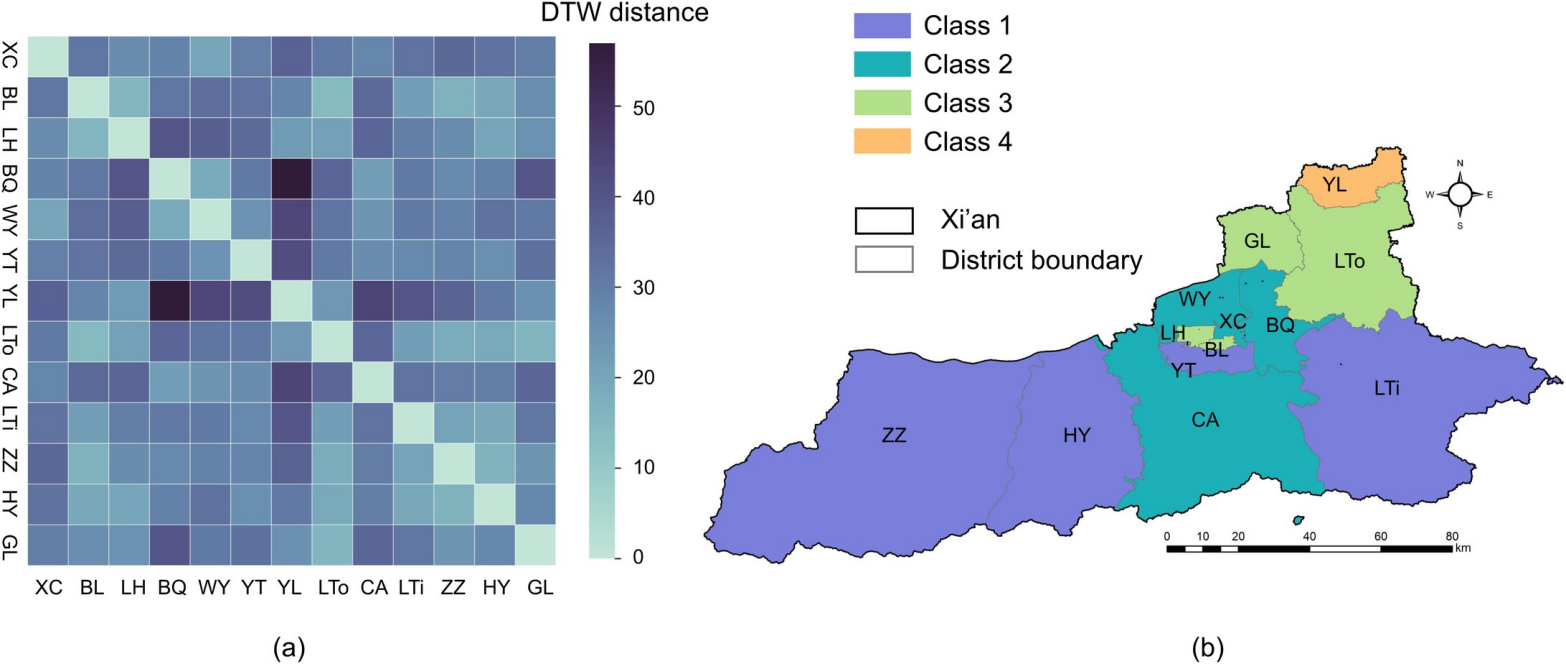
Similarity measurement of urbanization associations with HFRS and cluster analysis. (a) DTW distances between different counties and districts. (b) Four clusters of 13 counties and districts based on DTW distances. The figure was produced in ArcGIS 10.8 (ESRI, Redlands, CA, USA) using shape files representing Xi’an City which were obtained from the basic geographic database in National Catalogue Service For Geographic Information of China (https://www.webmap.cn/mapDataAction.do?method=forw&resType=5&storeId=2).

The similarity measurement and cluster analysis among the different districts of Xi’an is illustrated in Fig 7. Using the DTW distance between every two actual curves shown in Fig 6, we calculated the similarity level to characterize the different influential patterns of urbanization that affect the HFRS incidence. It is clear that a large part of Xi’an, including Zhouzhi, Huyi, Lantian, and Yanta districts, can be categorized into the first class characterized by a higher incidence of HFRS and least affected by rapid urbanization. The second class mainly covered Chang’an, Weiyang, Baqiao, and Xincheng districts located in the central city and southern to northern urban fringes and had a lower urbanization level than that of the first class but higher than that of the northeastern surrounding suburbs. This is because the rate of HFRS incidence in the second class was less affected by the urbanization processes. The third class consisted of Lianhu, Beilin, Gaoling, and Lintong districts containing both urban and suburban areas. This cluster is noticeable as it has peak turning points that correspond to its fitting curves, which suggests that these districts are still in the second phase of urbanization impacting the rate of HFRS incidence. The fourth class merely contained Yanliang District, which experienced the first phase of rapid urbanization that exacerbated the HFRS incidence, possibly because the flourishing aviation industry in this district can impede and slow down the rapid urbanization process to some extent.

### Spatiotemporal associations of the distribution of water bodies on HFRS incidence

**Fig 8:**
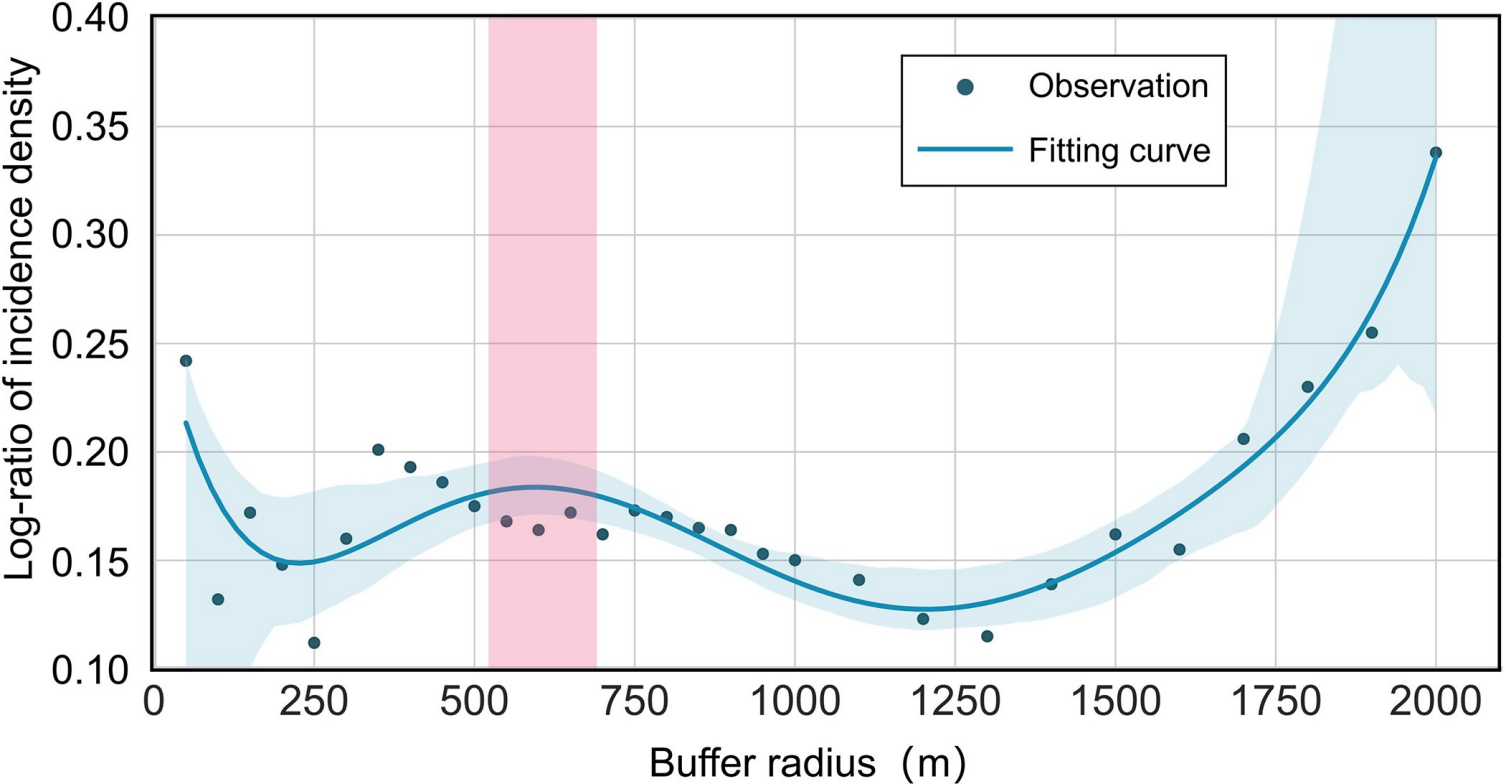
Logarithm fitting curve of the average annual density of HFRS cases within different radii.

The number of HFRS cases within different radii of water bodies was obtained for each year by performing the buffer analysis of water bodies in Xi’an from 2005 to 2018 (S4 Fig). The rate of HFRS incidence increased more dramatically within approximately 350 m and 675 m radii of the water bodies compared to that beyond these two radii. Hence, 350 m and 675 m can be preliminarily taken as the thresholds where the association of water bodies with the HFRS incidence rate may reach peak values. A high-order polynomial algorithm was realized to fit the weighted average of the HFRS incidence data, which yielded a fitting curve to indicate a critical point approximately 600 m away from the water bodies (Fig 8). The dramatic increase in the variation at the tail end of the logarithm fitting curve is possibly caused by a gradual decline in the number of HFRS cases with the buffer zone expansion [30].

**Fig 9:**
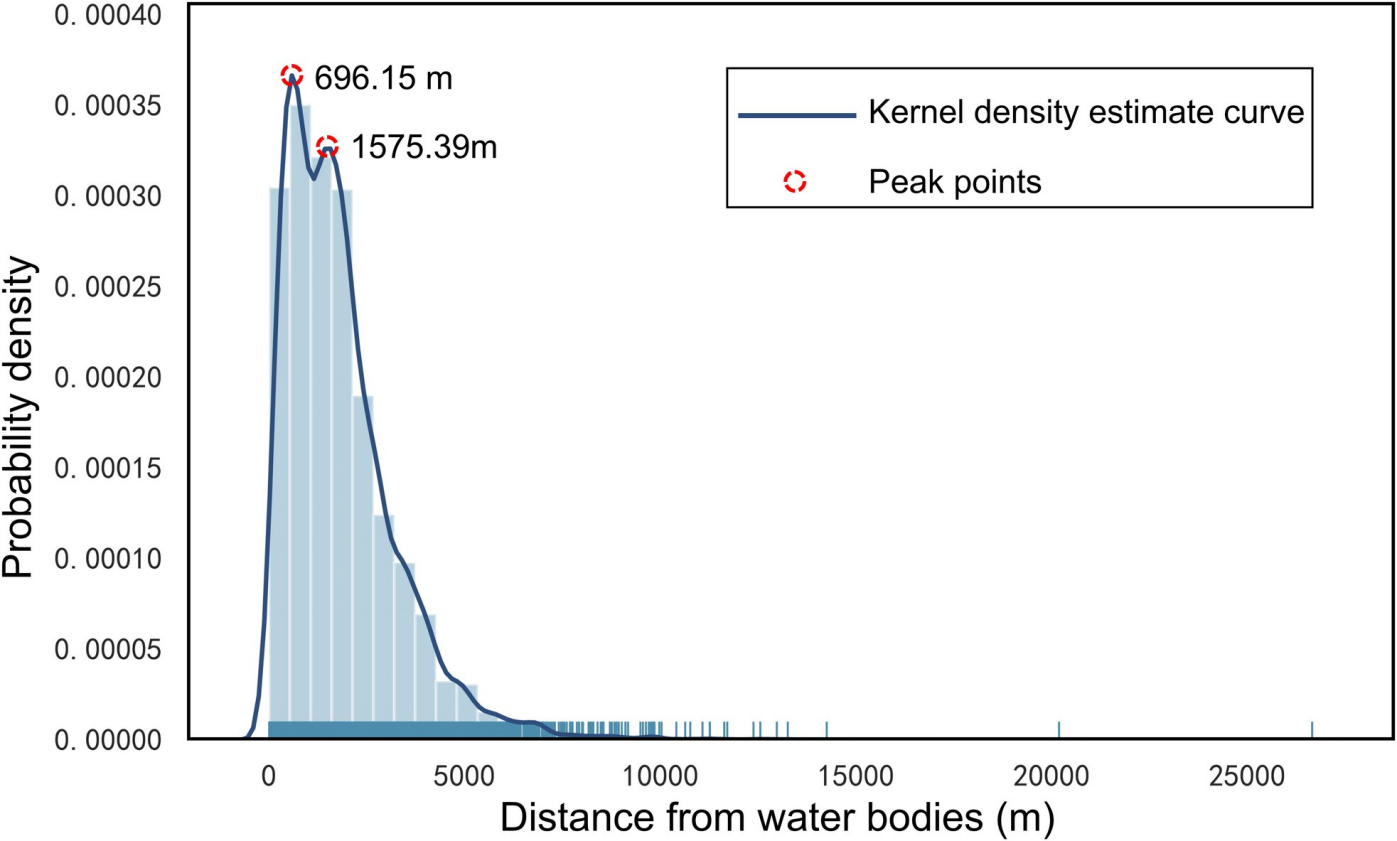
Kernel density estimate curve of the distance of HFRS cases from water bodies.

The KDE result in Fig 9 shows two peak points at approximately 696.15 m and 1575.39 m. The former one signifies the point of maximum probability. The probability density tended to increase till 696.15 m and then began to decrease after 1575.39 m, which means that there is a high probability that the area between these two peak points may show a high rate of HFRS incidence. The proportion of HFRS cases within 5 km of the water bodies was 96.45%, although several cases were also observed at >10 km from the water bodies. The main peak point of 696.15 m matches well with the threshold distance obtained from buffer analysis, which is approximately 600 m away from the water bodies. Since the buffer analysis primarily extracted peak points within 1 km, these two peaks obtained by the KDE curve are more likely to be the threshold distances to characterize the association between the distribution of water bodies and the HFRS incidence in Xi’an.

**Fig 10:**
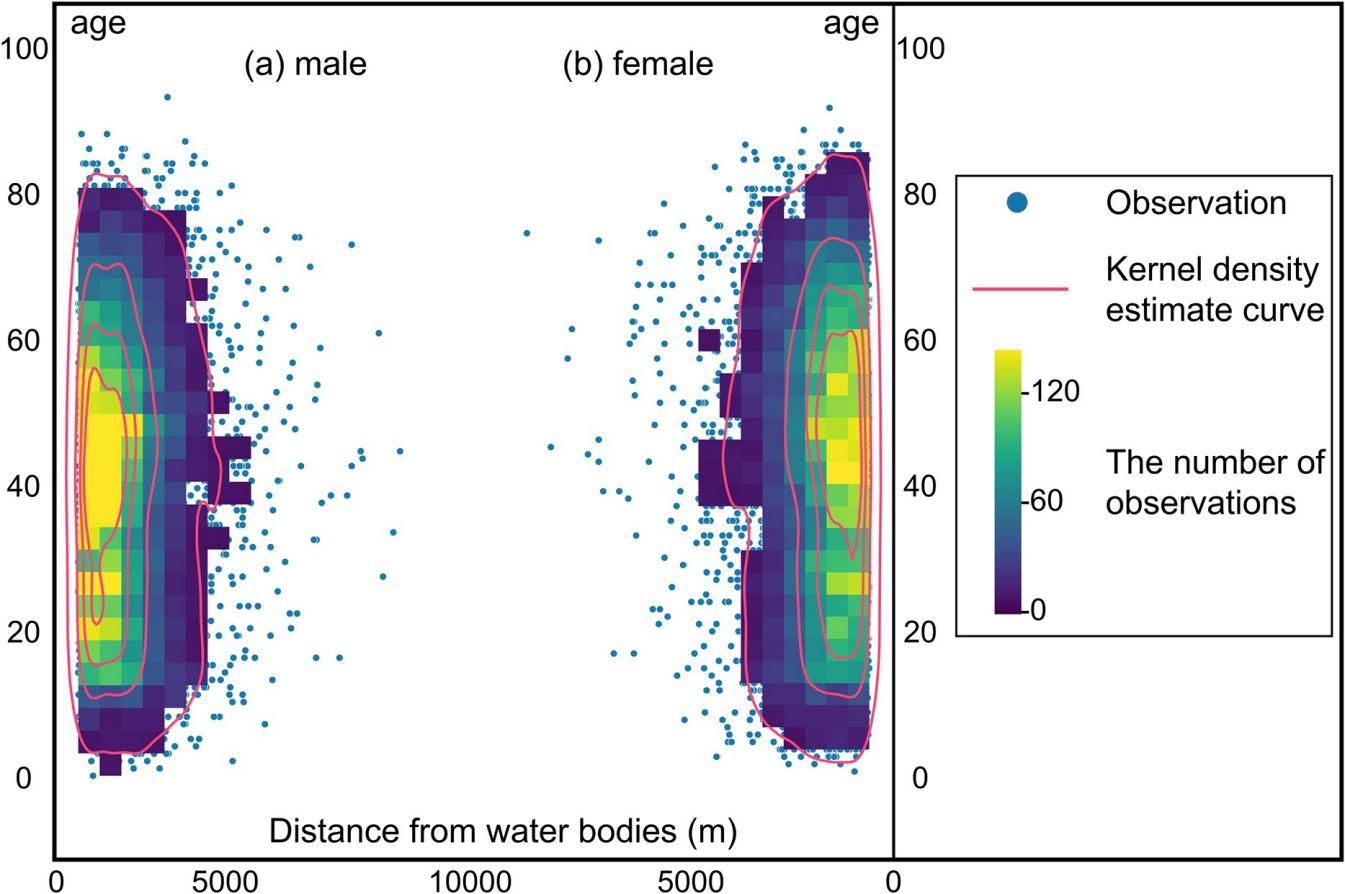
Bivariate distributions of the distance of HFRS cases from water bodies. The bivariate distributions are based on the age and sex of HFRS patients in Xi’an City.

**Fig 11:**
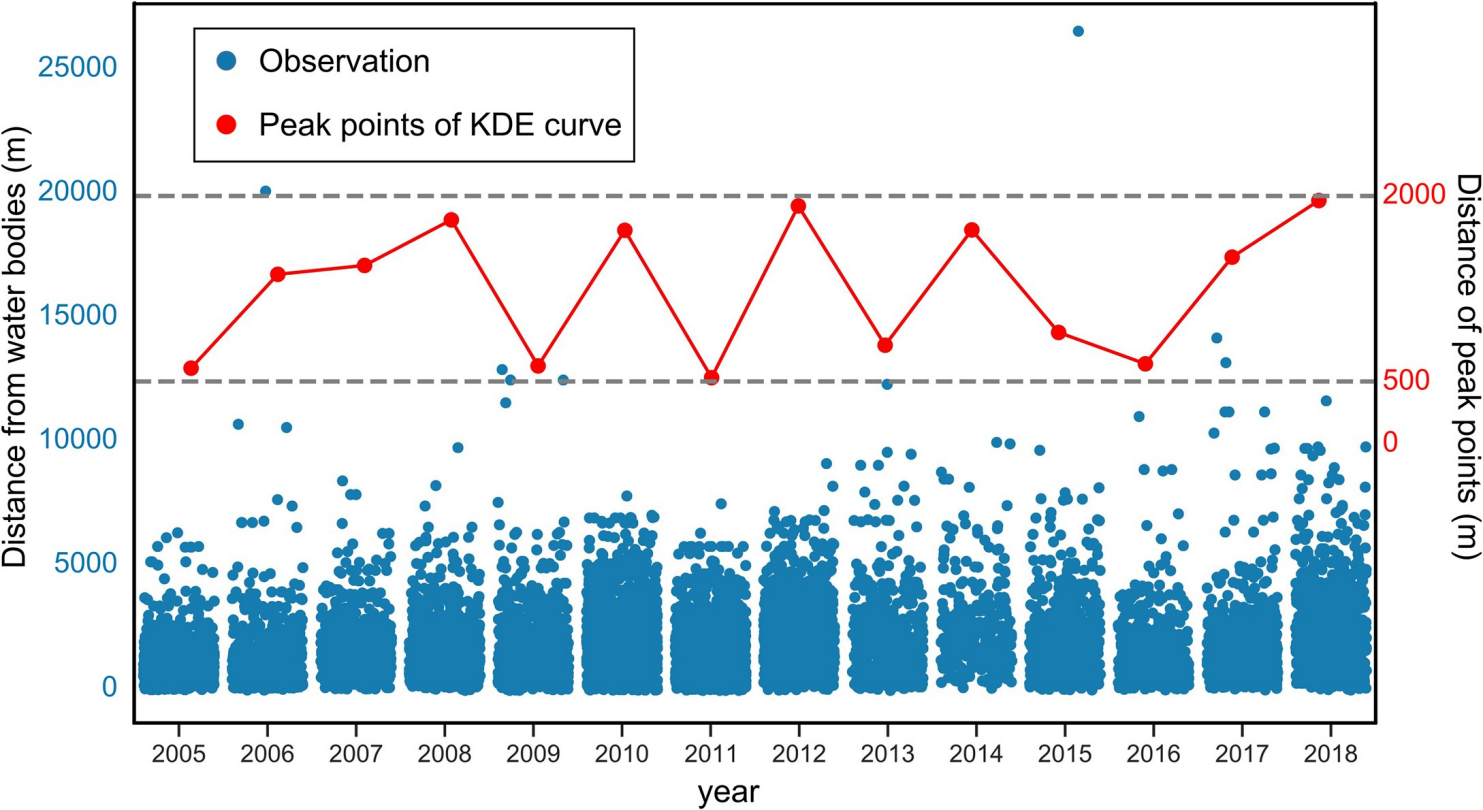
Dynamic change in the peak points of annual KDE curves. These curves reflect the distance of HFRS cases from water bodies in Xi’an City.

Two-dimensional KDE curves based on the age and sex of patients infected by HFRS in Xi’an are shown in Fig 10. No difference was observed in the rate of HFRS incidence based on the sex within 0–5 km of water bodies. Although the total number of female patients with HFRS was less than that of male patients, the average age of the former was generally higher than that of the latter. Hence, no significant difference in the spatial associations of water bodies can be found between HFRS cases based on the age and sex of patients. In addition, the time series data obtained from the annual KDE curve from 2005 to 2018 showed that all the peak points ranging from 500 m to 2000 m have clear periodic variations that generally move upward (Fig 11). Moreover, the peak point value reveals a noticeable increase after 2016, which implied a temporal increase in the spatial range of the association between the distribution of water bodies and the HFRS incidence in Xi’an.

### Interactions between urbanization and other factors

**Fig 12:**
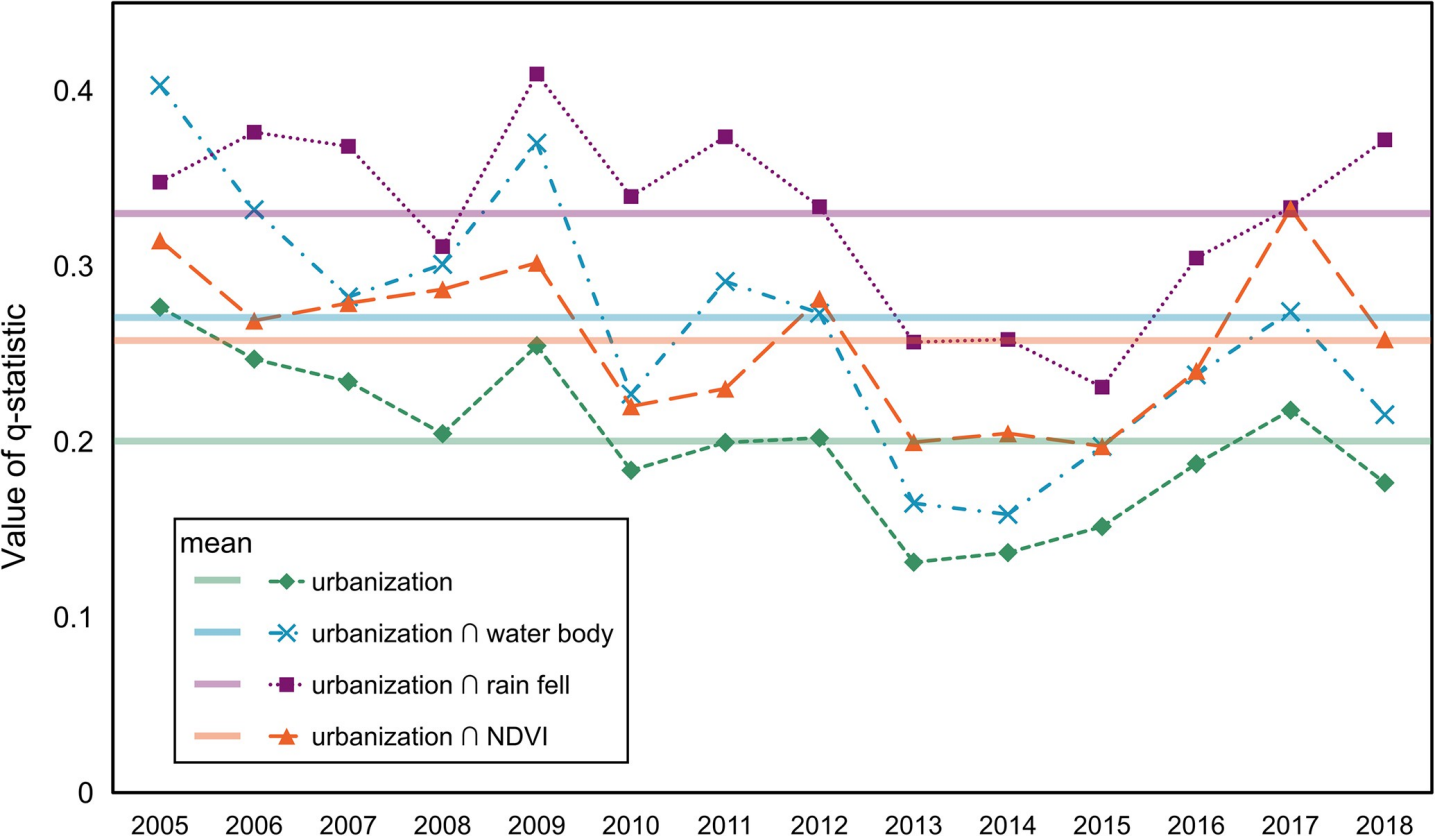
Interactions between urbanization, water body, rainfall, and NDVI.

The values of *q*-statistic in Fig 12 demonstrate the interactions between urbanization, water bodies, NDVI, and rainfall on the incidence of HFRS. The most significant relationship was that between urbanization and rainfall, indicating that rainfall is a significant climactic condition that can greatly affect the interactions between urbanization and HFRS incidence compared to the other two factors. Urbanization, when considered as an individual explanatory variable, plays an important role in the interactions with other factors and influences the HFRS incidence to a great extent. This observation was supported by the evident similarity among the variation trends of *q*-statistic values for urbanization and its interactions with other factors (Fig 12). Therefore, urbanization can be considered as the most critical factor affecting the HFRS incidence in regions experiencing rapid socioeconomic development such as Xi’an. At the same time, other factors such as rainfall, vegetation, and water bodies should also be taken into account for examining the spatiotemporal dynamics of HFRS.

**Fig 13:**
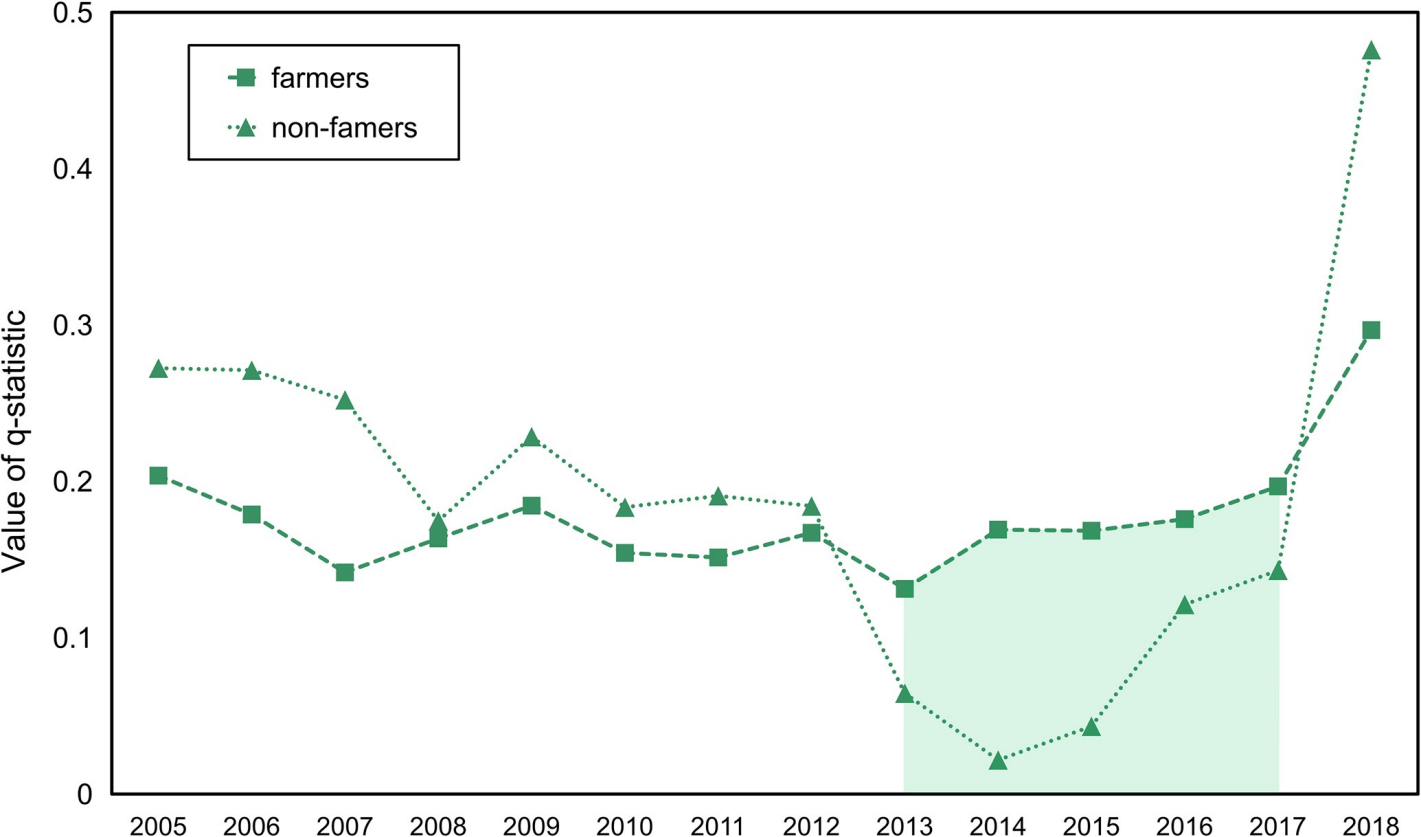
Relation between occupation type within the urbanized area and HFRS.

As over 65% of the patients with HFRS were farmers, we classified the HFRS patients into two general categories based on their occupation type: famers and non-famers. Non-farmers mainly comprised students and workers. Fig 13 shows the *q*-statistic values derived from analyzing the relationships between urbanization and HFRS cases in farmers and non-famers. The results show that with the exception of period from 2013 to 2017, non-famer HFRS patients had higher *q*-statistic values than that of the farmer HFRS patients. This exception was observed because the dramatic decrease in HFRS cases in non-farmers increased the lower response variable. Furthermore, Fig 13 demonstrates that a weaker relationship between farmer HFRS patients and urbanization than that between the non-famer HFRS patients and urbanization process. This phenomenon can be explained by the fact that the influence of urbanization on HFRS normally distributed in the rural–urban fringe areas rather than in the rural area far away from the urban core.

## Discussion

As an interdisciplinary research, this study attempts to investigate the spatiotemporal association of rapid urbanization and distribution of water bodies with the HFRS incidence in the city of Xi’an over a 14-year period from (2005–2018). Our results suggested at the presence of a statistically significant HFRS cluster in Xi’an over the entire study period. The majority of HFRS cases spatially concentrated in Chang’an District and the epidemic gradually spread to the surrounding area. Clusters of lower HFRS incidence mainly occurred in the northern districts, including Lintong, Gaoling, and Baqiao. These two clusters are different probably because of the different rodent host densities and host hantavirus-carrier rates, especially for the dominant rodent host *A*. *agrarius* commonly found in the Guanzhong Plain in the central Shaanxi Province [31]. The temporal variation in the HFRS incidence exhibited monthly fluctuations with two peak periods: June–July and November–January. It can be hypothesized that the peak HFRS incidence possibly occurs in the fall and winter, particularly in November. This result is in good agreement with those of previous studies [22,32]. Thus, our findings suggest that prevention and control measures such as HFRS vaccination should be reinforced before the peak time of HFRS incidence.

Our research also indicated that both rapid urbanization and distribution of water bodies can lead to a spatiotemporal association between the HFRS infection at different levels. As stated above, the association of rapid urbanization with the HFRS incidence has two different time phases. In the first phase, urbanization caused a gradual increase in the rate of the HFRS infection, while in the second, the association began to decrease to approach the average incidence rate of the previously urbanized area. This turning time point occurred in the sixth year of the study period characterized by rapid urbanization. We hypothesized that this phenomenon can be primarily explained by the fact that rapid urbanization dramatically facilitated the increase in the rodent host population and density, particularly in Zhouzhi, Huyi, Lantian, and Yanta districts of the first class. This is because rapid urbanization destroyed the natural habitat of the rodents and activity rules of animal reservoirs and severely altered their population distribution, thereby increasing the risk of exposure to anthropogenic circumstances. Moreover, the interactions between urbanization and other influential factors, including waterbodies, rainfall, and vegetation on the HFRS incidence should also be taken into consideration. An integration analysis of these factors can help better interpret the association between urbanization and HFRS dynamics. In addition, there is a significant difference in HFRS infection among people in distinct types of occupation, which possibly determines the distance of their living places or workplaces to the urbanized area. As the Government of China is promoting further urbanization by implementing various development strategies such as the Belt and Road Initiative, development of the Xi’an National Central City, and development of Guanzhong Plain urban agglomeration, the pace of urbanization in the Guanzhong Plain will further accelerate. As a result, Xi’an City will become even more vulnerable to endemic outbreaks of HFRS in the rapidly urbanizing pockets of the city with some possible local clusters. Therefore, policy makers should pay more attention to the targeted areas while allocating public health resources and services.

Furthermore, this work also revealed that the distribution of water bodies is an important driver of the spatiotemporal variations of HFRS incidence in Xi’an. Two significant thresholds of 696.15 m and 1575.39 m were observed, wherein the association of water bodies with the HFRS incidence rate in the surrounding areas peaked, especially at a distance of approximately 600 m away from the water bodies. Since water bodies play a critical role in changing the humidity and temperature that further affect the formation and maintenance of the host habitat, our study results may help in understanding how water-body distribution influences human exposure to rodents or their body fluids/excreta from a spatiotemporal perspective [33]. The methods of buffer analysis and KDE analysis used in this research may yield the same findings, suggesting that regions at approximately 600 m from the water bodies may have a high risk of Hantavirus infection and are almost unaffected by the sex or age of patients. Therefore, effective disease surveillance and seroprevalence investigation should be implemented in this region to reduce the HFRS incidence.

In addition, this study provides a practical example of how to apply geostatistical approaches to quantitatively explore the dynamic patterns, variations, and influential determinants of an epidemic, which not only demonstrate a potential way to address some research gaps in the existing literature, but also present several opportunities to support public health decision-makers in implementing effective strategies for preventing epidemics. However, the study has some limitations as well. Firstly, HFRS is a complicated communicable disease that can be co-influenced by multiple potential factors such as the rodent population, climate, topography, land-use types, and socioeconomic conditions. It is beyond the scope of the present study to investigate all these elements because of the lack of data on the complex mechanism of HFRS transmission. In spite of our preliminary analysis on the interactions between urbanization and distribution of water bodies, rainfall, and vegetation on the HFRS incidence, it is still a challenging task to further quantitatively reveal their comprehensive relationships based on a spatiotemporal perspective. Secondly, the mechanism of association between rapid urbanization and distribution of water bodies on the HFRS incidence can be interfered by the rodent host species. However, the access to data on rodent species is restricted by research funding, data availability, and time constraints. Thirdly, since urbanization typically takes a very long time spanning several years to show clear changes in satellite imagery, it is quite challenging to investigate the effects of urbanization on the monthly or seasonal HFRS incidence. In addition, it is difficult to obtain satellite imagery and statistical data for each month or season to match the data temporally for further investigation. Finally, the patients might be infected with the HV in or around their living places, farmlands, workplaces, or somewhere else, and it is difficult to determine the exact location where they got infected because of the limited information available from the acquired data. Therefore, we had to use the residential addresses of patients extracted from the available data in our current analysis. Nevertheless, the methodologies described in this study lay a preliminary foundation that can be extended to other regions as well at the national and even international levels for a wider application and precise interpretation of the results that may improve our understanding of various zoonoses.

## Conclusions

We demonstrated the application of both traditional statistical and geospatial methods to investigate and interpret how rapid urbanization, distribution of water bodies and other factors such as vegetation, rainfall, and occupation type are quantitatively and spatiotemporally associated with the rate of HFRS incidence in the city of Xi’an. Apart from the advantages for the research on epidemics, this study may also provide some useful decision support in planning and implementing various strategies for disease and reservoir surveillance, seroprevalence survey, diagnosis, and allocation of health care resources. Hence, this study is relevant to multiple stakeholders, including the general public, government, public health institutions, and volunteers, as it will provide them some useful insights to formulate an effective prevention and control strategy for HFRS.

## Ethics statement

In China, the collection of data from HFRS cases is part of routine public health surveillance, and such data collection is exempt from institutional review board assessment. Ethical approval for this study was not required in accordance with local legislation and national guidelines.

## Supporting information

S1 Eq Equations of entropy weights calculation. (DOCX)

S2 Eq Equation of the GeoDetector model. (DOCX)

S1 Table Global spatial autocorrelations of HFRS cases for Xi’an City, Northwestern China, from 2005 to 2018. (DOCX)

S1 Fig Study area: City of Xi’an, Shaanxi Province, Northwest China. The maps in the figure were produced in ArcGIS 10.8 (ESRI, Redlands, CA, USA) using shape files representing Xi’an City and China which were obtained from the basic geographic database in National Catalogue Service For Geographic Information of China (https://www.webmap.cn/mapDataAction.do?method=forw&resType=5&storeId=2).
(TIF)

S2 Fig The spatial dynamics of HFRS incidence in Xi’an for period 2005–2018 with the results of local autocorrelation analysis. The maps in the figure were produced in ArcGIS 10.8 (ESRI, Redlands, CA, USA) using shape files representing Xi’an City which were obtained from the basic geographic database in National Catalogue Service For Geographic Information of China (https://www.webmap.cn/mapDataAction.do?method=forw&resType=5&storeId=2).
(TIF)

S3 Fig Monthly temporal distribution of the average rate of HFRS incidence in Xi’an for period Jan 2005–Dec 2018. (TIF)

S4 Fig Number of HFRS cases within different radii of the water bodies for each year for Xi’an City from 2005 to 2018. (TIF)
