# Supplementary material for: Spatiotemporal association of rapid urbanization and water-body distribution on hemorrhagic fever with renal syndrome: A case study in the city of Xi’an, China
Source: PLoS Negl Trop Dis. 2022 Jan 10;16(1):e0010094. doi: 10.1371/journal.pntd.0010094 (PMC8782472; doi:10.1371/journal.pntd.0010094)
Supplement: S1 Eq — (DOCX) [file pntd.0010094.s001.docx]

**Equation 1 Equations of entropy weights calculation.**

Firstly, Considering the number of valid samples, the interval method is used to process the data (1), and the proportion of the *i-*th year in the *j*-th index was calculated (*m* = 14, *n* = 9) using Equation (2), and then the entropy of the *j*-th index was determined using Equation (3). Next, we acquired the difference coefficient of the *j*-th index using Equation (4) and the entropy weight coefficient of each index from Equation (5).

$X_{ij}=\frac{1}{1000}+10\frac{x_{ij}-x_{j\min}}{x_{j\max}-x_{j\min}}$ (1)

$P_{ij}=\frac{X_{ij}}{\sum_{i=1}^{m} X_{ij}}$ (2)

$e_{j}=-\frac{1}{\ln m}\sum_{i=1}^{m} P_{ij}\ln P_{ij}$ (3)

$g_{j}=1-e_{j}$ (4)

$w_{j}=\frac{g_{j}}{\sum_{j=1}^{n} g_{j}}$ (5)

*X_ij_* is the interval data, *x_ij_* is the initial value, *x_jmax_* is the maximum value of the *j*-th index in all years, and *x_jmin_* is the minimum value of the *j*-th index (i = 1, 2, ..., 14; j = 1, 2, ..., 9).
