# Supplementary material for: Spatiotemporal association of rapid urbanization and water-body distribution on hemorrhagic fever with renal syndrome: A case study in the city of Xi’an, China
Source: PLoS Negl Trop Dis. 2022 Jan 10;16(1):e0010094. doi: 10.1371/journal.pntd.0010094 (PMC8782472; doi:10.1371/journal.pntd.0010094)
Supplement: S2 Eq — (DOCX) [file pntd.0010094.s002.docx]

**Equation 2 Equation of the GeoDetector model**

$q=1-\frac{\sum_{h=1}^{L} N_{h}\sigma_{h}^{2}}{N\sigma^{2}}$ (1)

*N* and σ^2^ represent the number of units and the variance of *Y* in a study area, respectively. *X* and *Y* are categorical variables. The *q*-statistic measures both linear and nonlinear associations between *X* and *Y*; *q* = 0 indicates that there is no coupling between *X* and *Y*; *q* = 1 indicates that *X* explains 100% of *Y*.
