# Supplementary material for: Spatiotemporal association of rapid urbanization and water-body distribution on hemorrhagic fever with renal syndrome: A case study in the city of Xi’an, China
Source: PLoS Negl Trop Dis. 2022 Jan 10;16(1):e0010094. doi: 10.1371/journal.pntd.0010094 (PMC8782472; doi:10.1371/journal.pntd.0010094)
Supplement: S1 Table — (DOCX) [file pntd.0010094.s003.docx]

**S1 Table. Global spatial autocorrelations of HFRS cases for Xi'an City, Northwestern China, from 2005 to 2018.**

| **Year** | **Moran's I Index** | **Moran's I Z-score** | **Moran's I *P*-value** | **Global spatial pattern** |
| --- | --- | --- | --- | --- |
| 2005 | 0.15 | 11.59 | <0.001 | Clustered |
| 2006 | 0.16 | 11.83 | <0.001 | Clustered |
| 2007 | 0.08 | 6.21 | <0.001 | Clustered |
| 2008 | 0.05 | 5.18 | <0.001 | Clustered |
| 2009 | 0.07 | 5.83 | <0.001 | Clustered |
| 2010 | 0.15 | 11.83 | <0.001 | Clustered |
| 2011 | 0.11 | 8.31 | <0.001 | Clustered |
| 2012 | 0.10 | 7.97 | <0.001 | Clustered |
| 2013 | 0.06 | 5.03 | <0.001 | Clustered |
| 2014 | 0.04 | 3.03 | 0.002 | Random |
| 2015 | 0.12 | 9.17 | <0.001 | Clustered |
| 2016 | 0.13 | 9.50 | <0.001 | Clustered |
| 2017 | 0.08 | 6.25 | <0.001 | Clustered |
| 2018 | 0.17 | 12.70 | <0.001 | Clustered |
