# Supplementary figures and images for: Spatiotemporal association of rapid urbanization and water-body distribution on hemorrhagic fever with renal syndrome: A case study in the city of Xi’an, China
Source: PLoS Negl Trop Dis. 2022 Jan 10;16(1):e0010094. doi: 10.1371/journal.pntd.0010094 (PMC8782472; doi:10.1371/journal.pntd.0010094)

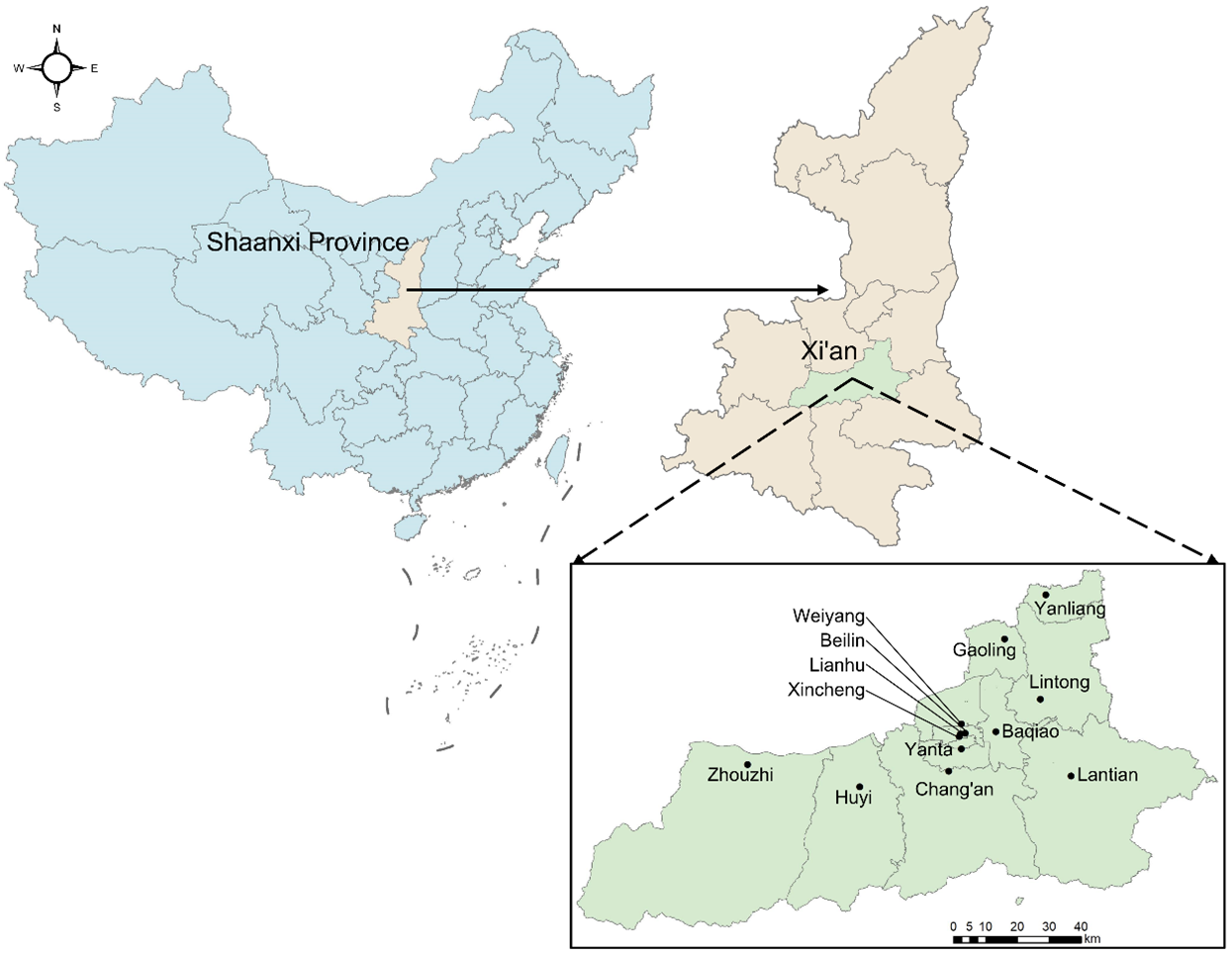

Supplement: S1 Fig — The maps in the figure were produced in ArcGIS 10.8 (ESRI, Redlands, CA, USA) using shape files representing Xi’an City and China which were obtained from the basic geographic database in National Catalogue Service For Geographic Information of China (https://www.webmap.cn/mapDataAction.do?method=forw&resType=5&storeId=2). (TIF) [file pntd.0010094.s004.tif]

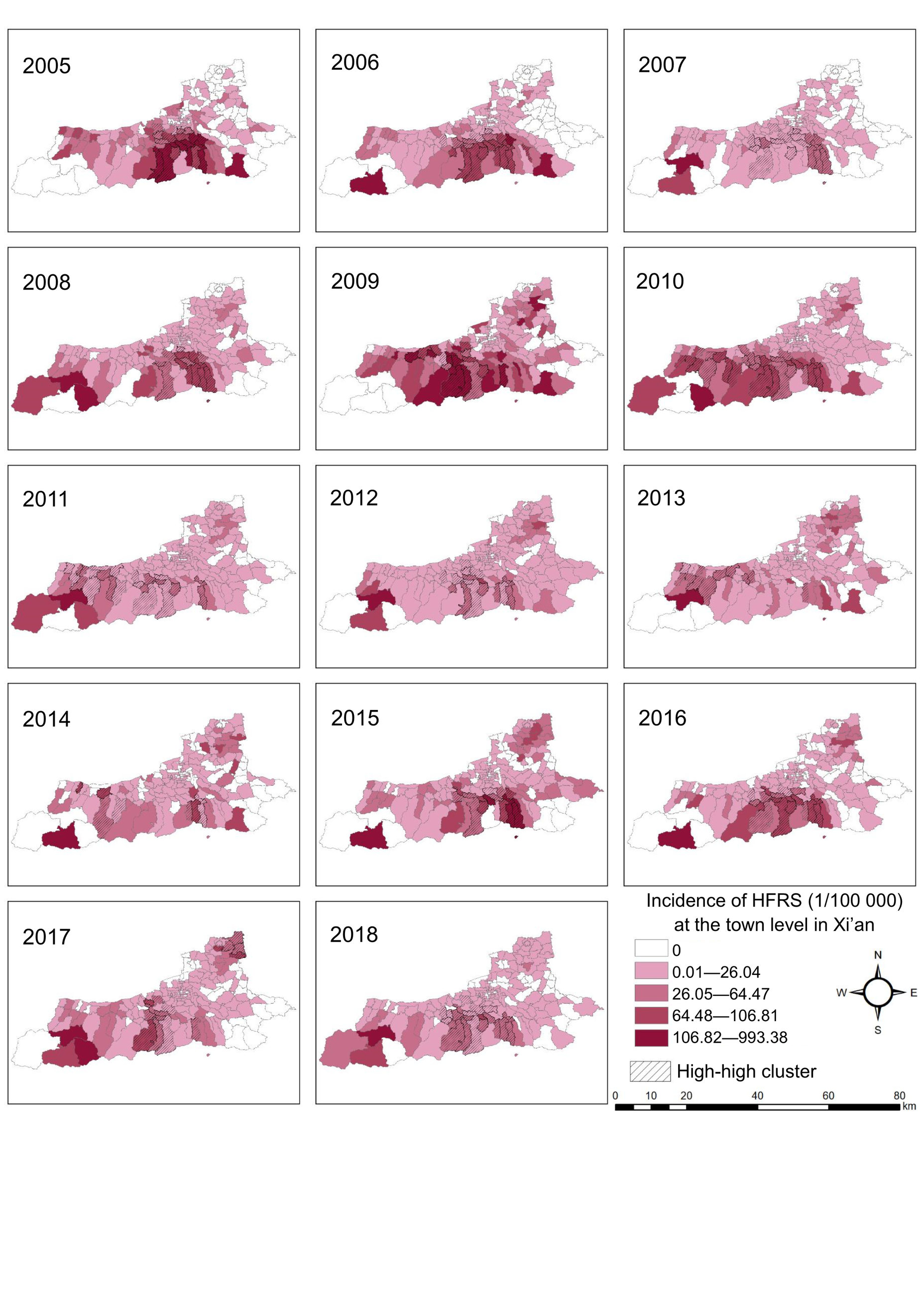

Supplement: S2 Fig — The maps in the figure were produced in ArcGIS 10.8 (ESRI, Redlands, CA, USA) using shape files representing Xi’an City which were obtained from the basic geographic database in National Catalogue Service For Geographic Information of China (https://www.webmap.cn/mapDataAction.do?method=forw&resType=5&storeId=2). (TIF) [file pntd.0010094.s005.tif]

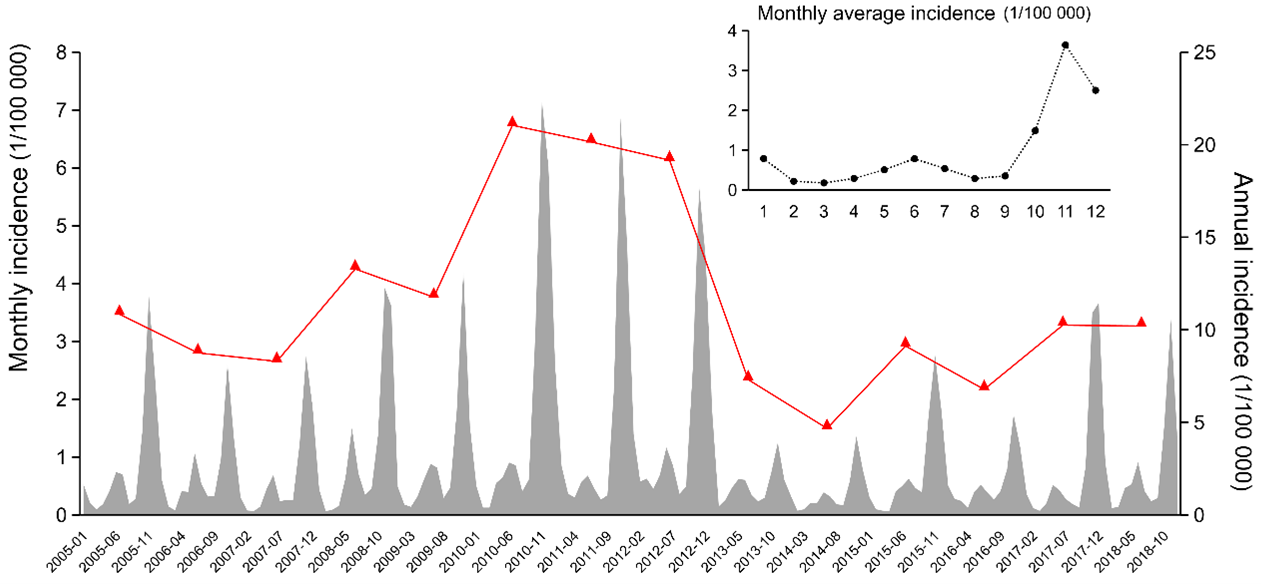

Supplement: S3 Fig — (TIF) [file pntd.0010094.s006.tif]

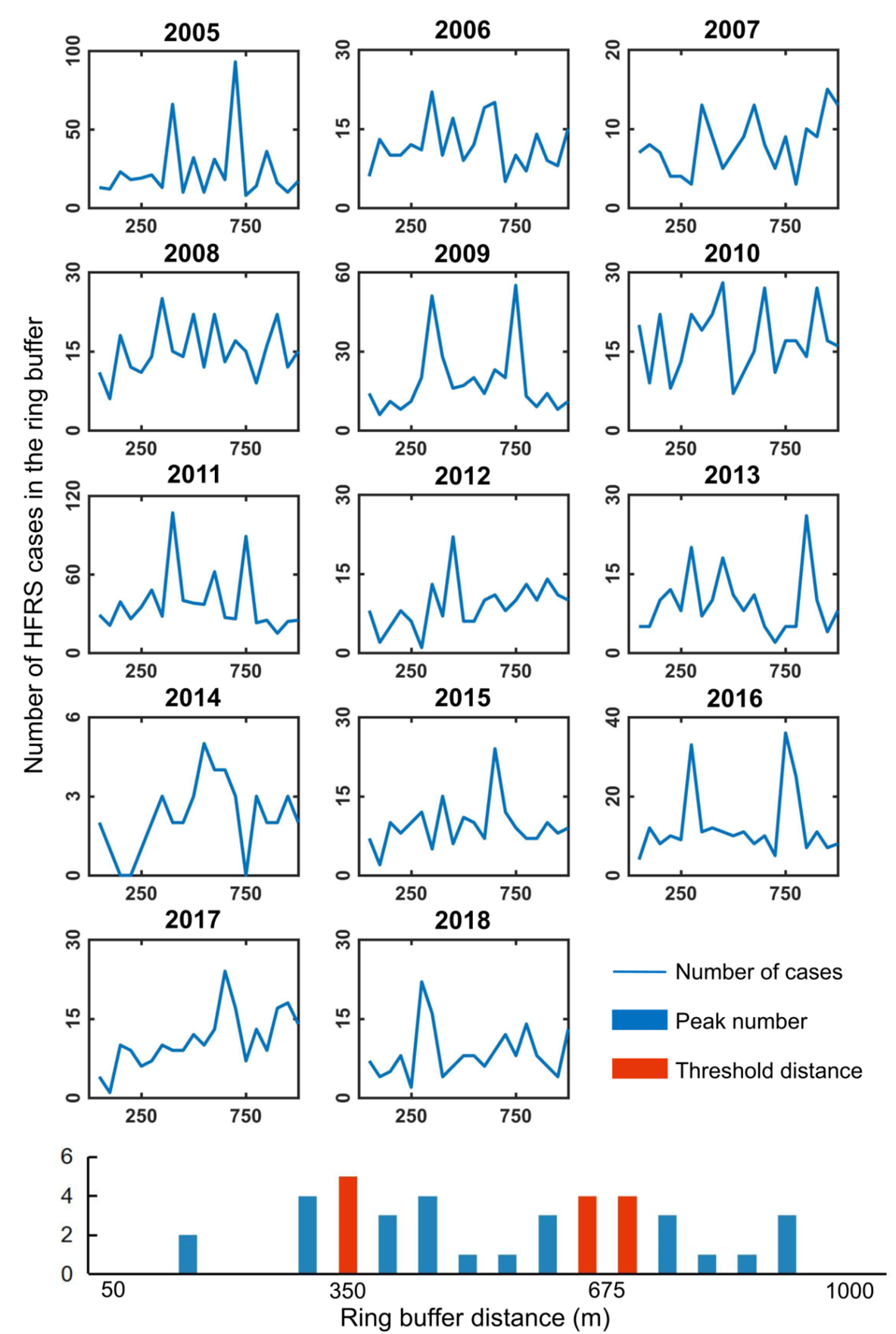

Supplement: S4 Fig — (TIF) [file pntd.0010094.s007.tif]
